# Antigenotoxicity and Tumor Growing Inhibition by Leafy *Brassica carinata* and Sinigrin

**DOI:** 10.3390/molecules200915748

**Published:** 2015-08-28

**Authors:** María-Dolores Lozano-Baena, Inmaculada Tasset, Sara Obregón-Cano, Antonio de Haro-Bailon, Andrés Muñoz-Serrano, Ángeles Alonso-Moraga

**Affiliations:** 1 Department of Plant Breeding, Institute of Sustainable Agriculture, CSIC, Avd. Menendez Pidal s/n, Córdoba E-14004, Spain; E-Mails: mdlozano@ias.csic.es (M.-D.L.-B.); saraobregon@ias.csic.es (S.O.-C.); 2 Department of Developmental and Molecular Biology, Chanin Building, Albert Einstein College of Medicine, 1300 Morris Park Avenue, Bronx, NY 10461, USA; E-Mail: b72tacui@uco.es; 3 Department of Genetics, Gregor Mendel Building, University of Córdoba, Rabanales, Córdoba 14014, Spain; E-Mails: ge1ams@uco.es (A.M.-S.); ge1almoa@uco.es (A.A.-M.)

**Keywords:** *Drosophila melanogaster*, HL60, antigenotoxicity, cytotoxicity, *Brassica*, sinigrin, bioactive compound

## Abstract

Cruciferous vegetables are well known and worldwide consumed due to their health benefits and cancer prevention properties. As a desirable cruciferous plant, Ethiopian mustard (*Brassica carinata* A. Braun) and its glucosinolate sinigrin were tested in the *in vivo Drosophila melanogaster* (SMART) and the *in vitro* HL60 (human promyelocytic leukaemia cell line) systems. High performance liquid chromatography (HPLC) analysis of plant samples confirmed the presence of sinigrin as principal *B. carinata* glucosinolate. SMART was performed by feeding *D. melanogaster* larvae either with different concentrations of plant/compound samples or combining them with hydrogen peroxide (a potent oxidative mutagen) being both antimutagenics. HL60 assays showed the tumoricidal activity of plant samples (IC_50_ = 0.28 mg·mL^−1^) and the breakdown products of sinigrin hydrolysis (IC_50_ = 2.71 µM). Our results enhance the potential of *B. carinata* as health promoter and chemopreventive in both systems and the leading role of sinigrin in these effects.

## 1. Introduction

There is a wide range of variation with respect to the properties of the botanical products used by humans. The active principles of vegetables constitute nowadays the basis of most pharmacological substances. In this sense, consumers not only appreciate vegetables for their nutritional value, but also for their contents in compounds that produce benefits for human health. For this reason, the analysis of the possible antimutagenic, anticarcinogenic and anti-aging activity of a botanical nutraceutical substance is essential as a tool to distinguish between medicines and simply healthy products.

Many beneficial properties have been attributed to cruciferous vegetables (*i.e.*, broccoli, cabbage, Brussels sprouts and cauliflower) [1,2]. Specifically, numerous members of the *Brassicaceae* family are commercialized for animal and human consumption around the world as a rich source of nutrients and also, as healthy products [3].

Glucosinolates (β-thioglucoside-*N*-hydroxysulphates, GLSs) are naturally occurring thioglucosides that are characteristic of the *Brassicaceae* (including the genus Brassica) and related family in the order *Capparales* [4]. These compounds remain inactive until hydrolysed to numerous compounds (thiocyanates, thiones, indoles, nitriles, *etc.*) which possess diverse biological activities as biocides by myrosinase enzymes (thioglucosidase) [5,6]. They are also known to possess anti-nutritional properties [7] although produce different effects in humans in function of their chemical structure and concentration in the plant. Both vary considerably depending on the species, stage of development, type of tissue and environmental conditions, making the determination of harmful and beneficial effects in human nutrition a difficult task [3].

*Brassica carinata* A. Braun, commonly known as Ethiopian mustard or Abyssinian mustard, belongs to the *Brassicaceae* family and is a traditional African vegetable, previously gathered from the wild for human consumption. It is cultivated as an oil and leafy vegetable plant in the Ethiopian highlands at altitudes between 1500 and 2600 m. It is known as yabesha gomen in Amharic and also used in East and Southern Africa as accompaniment for ugali (a type of porridge made from maize or millet flour) [8,9]. *B. carinata* is an annual plant with many desirable traits for commercialization: high yielding and rusticity, edible leaves, resistance to disease and low chemical input requirement [10,11]. The plant may be eaten whole and possess a higher nutrient composition than other dietary species like white cabbage and spinach [12].

The predominant GLS in *B. carinata* is sinigrin, but its concentration depends on different factors, such as genotype, tissues and plant age [13]. Sinigrin hydrolysis catalyzed by myrosinase enzymes produces isothiocyanates (ITCs) as bioactive products [14,15], specifically allyl-ITCs (AITC) whose anticancer activity has been proved by several authors [16,17,18]. It has been confirmed that even short-term intake of ITC-containing vegetables might be associated with reduced cancer risk in human *in vitro* and *in vivo* systems [19].

Epidemiological studies highlight that the present consumption rate of this vegetables has benefits for human health even more than diets rich in fruits and other vegetables [20]. However, *in vivo* and *in vitro* experiments show variable results depending on the species, and GLS type and content seem to be the key. The present work is based on the need to clarify the balance of adverse and beneficial effects of cruciferous plants already selected for their desirable traits. This is the case of tested plant, the leaves of *B. carinata*, which has been used to determine the antimutagenic and antiproliferative capacities of plant samples and its major GLS sinigrin, as well as the concentrations and time at which their health benefit appears. The *Drosophila melanogaster in vivo* animal model and the HL60 *in vitro* cell-line model were used for these two purposes.

## 2. Results and Discussion

Depending on nutrient content, vegetables are categorized into five groups and cruciferous plants fall into two of them [21]: the dark-green and the other vegetables categories, being the dark-green one of the main recommended group for human intake. Information about vegetables and diet, including how much of these foods should be eaten daily or weekly is available from the U.S. Department of Agriculture (USDA) website Choose My Plate.

Due to the large variety among cruciferous species and the high variability of their compound content, the daily human intake of *Brassica* sp. and GLSs is difficult to estimate. During the past decades, commercial varieties of broccoli have been consumed as most popular *Brassica* species in a range of 2–8 grams per day around the world, much less than those recommended for total vegetable intake [6]. Also, the corresponding daily GLSs intake in humans is very variable depending on countries and populations, as well as consumed varieties, but it has been estimated to be in the order of milligrams [22]. Despite the great amount of works and studies about this topic, nowadays there are no specific recommendations for only *Brassica* intake apart from those for general vegetables. However, a current intake assessment conducted by Spherix indicated that the mean and 90th percentile consumption of 16.4 and 50.9 grams per day, respectively, is less than one serving of vegetables (one serving of raw broccoli is 36 g; one serving of cooked broccoli is 78 g [23]. Taking into account the average daily food intake of *D. melanogaster* (between three to five times its own weight) [24] and its average body weight (1 mg) [25], the concentration ranges assayed for both *B. carinata* and sinigrin falls within this consumption rate.

### 2.1. Leaf Glucosinolate Content Determination

**Figure 1:**
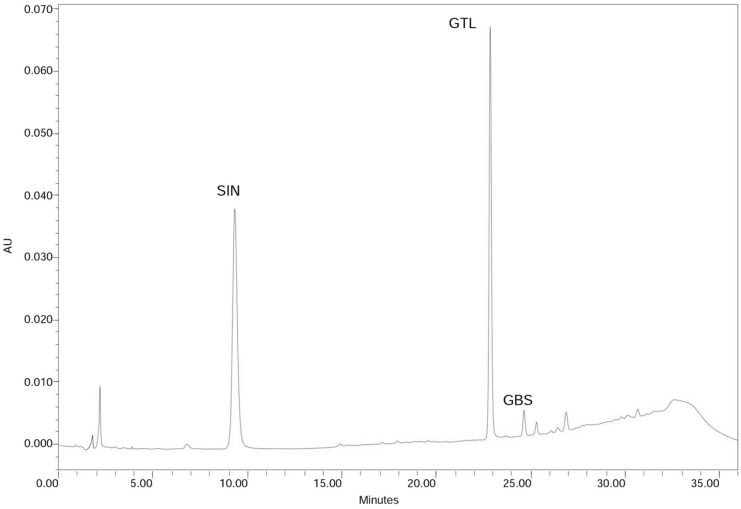
Chromatogram of *B. carinata* leaf samples. Peaks correspond to the identified GLSs: sinigrin (SIN) and glucobrassicin (GBS), and the internal standard glucotropaeolin (GTL).

The HPLC chromatogram of the analysed leaf samples is shown in Figure 1. Only two GLSs were detected: sinigrin (2-propenyl-GLS) (10.05 µmol·g^−1^ of dry weight, DW) and glucobrassicin (3-indolylmethyl-GLS), the last one in small amounts (0.14 µmol·g^−1^ DW). The total GLS content found in the leaves of the *B. carinata* line (Bc-IASC1) was 10.19 µmol·g^−1^ DW (equivalent to 101.9 µmol per 100 g fresh weight). Our data for GLS and sinigrin content in *B. carinata* are in accordance with previous determination in leaves at the same growth stage and resulting sinigrin the major and almost sole GLS presented [13].

### 2.2. In Vivo Assays

#### 2.2.1. Toxicity Assays

Table 1 shows *B. carinata* plant and sinigrin sample toxicity expressed as percentage of emerged treated adults respect to the negative control emerged adults (survival control corrected). All treatments at all assayed concentrations affected *D. melanogaster* survival with values between 19.33% and 78.22%, both for sinigrin 1.2 mM in combined and simple treatments respectively. Simple treatments produced a larvae survival around 50% (LC_50_), which represents normal value in experiments performed to evaluate toxicity levels.

**Table 1:** Toxicity levels of *B. carinata* and sinigrin in SMART expressed as *Drosophila* adults survival after simple and combined treatment (without and with H_2_O_2_ respectively).

Toxicity							
Survival ^a^ % Treatments							
Controls	Simple	*B. carinata* (mg·mL^−1^)	Simple	Combined ^b^	Sinigrin (mM ^c^)	Simple	Combined ^b^
H_2_O	100	1.25	55.33 *	37.78 *	0.60	56.44 *	25.56 *
H_2_O_2_ (0.12 M)	37.78 *	2.50	53.33 *	30 *	1.20	78.22 *	19.33 *
		5.00	47.56 *	36.89 *	2.40	61.56 *	47.33 *
					4.81	76.89 *	60.22 *
Average			52.07	34.89		68.27	38.11

^a^ Survival expressed in percentage as emerged adult total of each treatment with respect to H_2_O control emerged adult total; ^b^ Combined treatments using standard medium and 0.12 M H_2_O_2_; ^c^ 1 mM sinigrin = 397.46 µg·mL^−1^ sinigrin; * Asterisks indicate significance levels with respect to the untreated control group (*p* ≤ 0.05).

Our results are in agreement with those found before [26] in which ITCs were toxic for coleopteran eggs, showing that lethal concentration (LC_90_) of these compounds were 1.8 to 5.6 µg·mL^−1^ depending on ITC type, being AITC less toxic than aromatic-ITCs.

Regarding *B. carinata* plant samples, the survival average was 52.07% and 34.89% for simple and combined treatments, respectively. This reduction of survival (~18%) is explained by the addition of H_2_O_2_ to the medium (combined treatment) as a potent toxic agent. This H_2_O_2_ effect was also observed in sinigrin treatments were survival average was 38.11% in combined against 68.27% simple treatments (~30% survival reduction).

In simple treatments, sinigrin showed a less toxic effect than *B. carinata* samples at all tested concentrations. This fact could be explained by the complex composition of plant samples when compared with single compounds. In this sense, *B. carinata* plant possesses anti-nutrients such as phytates, phenolics, and tannins which can affect individual survival [3,5].

In combined treatments, *B. carinata* addition had no significant effect on *D. melanogaster* survival so that the number of emerged adults was similar to those of positive control (H_2_O_2_ treatment survival = 37.78%) although some synergistic but not additive effect of H_2_O_2_ and *B. carinata* products is found in concentrations of 2.5 and 5 mg·mL^−1^. Contrarily, sinigrin addition to the medium counteracted H_2_O_2_ toxicity at the highest assayed concentrations (2.4 and 4.81 mM) but, this effect of detoxification did not appeared at low sinigrin concentrations (0.6 and 1.2) in which the toxic effect was higher, reducing the number of emerged adults (25.56% and 19.33% of survival, respectively).

In general, no dose effect was observed except in sinigrin combined treatments in which the lesser survival percentage (19.33%) corresponded to a concentration of 1.2 mM and the highest percentage (60.22%) to the highest concentration assayed (4.81 mM). Other concentrations could be assayed in order to determine a dose effects in tested samples but they would not be relevant for human consumption.

#### 2.2.2. Genotoxicity Assays

Direct studies on the toxicity and genotoxicity of *B. carinata* fresh plant are not available because the epidemiological data always shows protective anticancer health properties [27]. The present study on the genotoxicity of *B. carinata* is the first that has confirmed the epidemiological references probing that *B. carinata* plant did not exert any DNA damage in the *mwh*/*flr* eukaryotic system of *D. melanogaster*.

Due to the antioxidant capacity of *B. carinata* [28] and sinigrin [29], H_2_O_2_ has been selected as oxygen free radical generator in this work. This compound has been showed as a potent mutagen [30] by producing highly reactive hydroxyl radicals (^●^OH). Thus, the present work has tried to prove the antioxidant capacity of tested samples which could act as antimutagens avoiding DNA damage produced by H_2_O_2_.

Table 2 shows the results of H_2_O_2_ genotoxic activity, as a positive control, both in marker-heterozygous and in balancer-heterozygous wings. H_2_O_2_ has been selected as a positive control due to its proved oxidative genotoxic activity in the SMART being able to induce somatic mutation and mitotic recombination [31]. Our results are consistent with this showing that H_2_O_2_ increased small single and total spots. The genotoxic results for H_2_O_2_ validate the assay as an appropriate system for screening mutagens (positive controls as H_2_O_2_) and non-mutagens (distilled-sterile water controls or safe plants).

The genotoxicity results obtained in the SMART of *D. melanogaster* for *B. carinata* and sinigrin are summarized in Table 2. No sample was mutagenic at any tested concentration. Contrarily they produce total mutation rates lower than those of the negative control at any dose, with an average of 0.18 and 0.15 spots per wing in the *B. carinata* and sinigrin experiments respectively. In this sense, food metabolism products generate free radicals in cells such as oxygen and nitrogen reactive species [32] which produce mutations. In SMART, these mutations are scored in negative control and they are due to larva feeding of standard Carolina medium. Any addition to this medium can increase (*i.e.*, the mutagen used, H_2_O_2_) or decrease this mutation range (free radical scavenger) which is the case of assayed samples, so our plant and molecule samples produce a mutation rate lower than negative control, acting as antimutagens against larva medium.

**Table 2:** Genotoxicity of *B. carinata* and sinigrin in SMART. Frequencies of mutations (spots/wing) for each category (Small, Large, Twin and Total) obtained in simple treatment.

Genotoxicity					
**Mutation Rate (Spots per Wing) Diagnosis ^a^**					
Treatment	N° of wings	Small single spots 1–2 cells; *m* = 2	Large single spots > 2 cells; *m* = 5	Twin spots *m* = 5	Total spots *m* = 2
**Controls**					
H_2_O	212	0.26 (54)	0.04 (8)	0.03 (5)	0.32 (67)
H_2_O_2_ (0.12 M)	168	0.60 (94) +	0.07 (11) −	0.06 (4) −	0.65 (109) +
**Plant Material: *Brassica carinata* (mg·mL^−1^)**					
1.25	40	0.01 (4) −	0.03 (1) −	0.03 (1) −	0.15 (6) −
2.50	48	0.15 (7) −	0.04 (2) −	0.02 (1) −	0.21 (10) −
5.00	48	0.13 (6) −	0.02 (1) −	0.02 (1) −	0.19 (8) −
**Single Compound: Sinigrin (mM) ^b^**					
0.60	40	0.18 (7) −	0.03 (1) −	0	0.20 (8) −
1.20	34	0.12 (4) −	0.03 (1) −	0.03 (1) −	0.18 (6) −
2.40	36	0.08 (3) −	0.03 (1) −	0	0.11 (4) −
4.81	28	0.14 (4) −	0	0	0.14 (4) −

^a^ Statistical diagnoses [33,34]: + (positive) and − (negative). Significance levels α = β = 0.05, one-sided test without Bonferroni correction; ^b^ 1 mM sinigrin = 397.46 µg·mL^−1^ sinigrin.

This non-genotoxic effect found in *D. melanogaster* are useful data to consider the inclusion of *B. carinata* in human diet even more than other *Brassica* spp. more popular and worldwide consumed *i.e.*, broccoli (*B. oleracea* L. var. *italica*) which raw, freeze-dried market plants lead to an increase in genotoxicity in this *in vivo* test [35]. In this sense, some recent studies gave rise to the concern that broccoli and other commercial varieties possess genotoxic activity due to their content in DNA damaging constituents such as certain types of GLSs [6,36]. Contrarily, we have not found this undesirable effect in our tested plant *B. carinata*, a fact that would reinforce the proposal for using it as a dietary source and makes this species more appropriate for human intake than its close relatives. The different GLS profiles found in *Brassica* spp. may be the responsible for this apparent discrepancy, as sinigrin is a minor GLS constituent in such a species [37] and contrarily, it is the main GLS in *B. carinata*.

Our results on the lack of genotoxicity of sinigrin are in concordance with previous studies [38] showing that sinigrin is neither genotoxic nor cytotoxic in the *in vitro* hamster ovary cell line (CHO) system. These authors also showed that AITC are not genotoxic at high cytotoxic doses as opposed to phenethyl-ITCs (breakdown products containing an aromatic functional group). Nevertheless, sinigrin induced chromosome aberrations but not sister chromatid exchanges at concentrations of 2 mg·mL^−1^. On the other hand, toxicity tests performed in the nematode *Caenorhabditis elegans* [14] concluded that sinigrin is non-toxic up to the concentration 80 g·L^−1^ and the addition of myrosinase increased sinigrin toxicity (LC_50_ = 0.5 g·L^−1^). The same experiment performed directly with AITC resulted in a lethal concentration value of 0.04 g·L^−1^.

#### 2.2.3. Antigenotoxicity Assays

The results obtained for antigenotoxicity assays are a contribution to the health properties of *B. carinata* and sinigrin in DNA protection. Analysed samples showed that this plant behaves as a desmutagen by reducing the apparition of mutations in comparison with negative control. Table 3 show the percentage of inhibition of *B. carinata* and sinigrin (respectively) when are assayed against H_2_O_2_. As expected, the addition of plant/compound samples to the fly food produced antimutagenic effects.

**Table 3:** Antigenotoxicity of *B. carinata* and sinigrin in SMART. Frequencies of mutations (spots/wing) for each category (Small, Large, Twin and Total) obtained in combined treatments.

Antigenotoxicity					
**Mutation Rate (Spots per Wing) Diagnosis ^a^**					
Treatment	N° of wings	Small single spots 1–2 cells; *m* = 2	Large single spots > 2 cells; *m* = 5	Twin spots *m* = 5	Total spots *m* = 2
**Controls**					
H_2_O	212	0.26 (54)	0.04 (8)	0.03 (5)	0.32 (67)
H_2_O_2_ (0.12 M)	168	0.60 (94) +	0.07 (11) −	0.06 (4) −	0.65 (109) +
**Plant Material: *Brassica carinata* (mg·mL^−1^)**					
1.25	40	0.11 (4) −	0.03 (1) −	0	0.14 (5) −
2.50	48	0.25 (7) −	0.04 (1) −	0	0.29 (8) −
5.00	48	0.27 (8) −	0.03 (1)	0	0.30 (9) −
**Single Compound: Sinigrin (mM) ^b^**					
0.60	40	0.19 (6) −	0	0	0.19 (6) −
1.20	34	0.20 (4) −	0	0	0.20 (4) −
2.40	36	0.25 (1) −	0	0	0.25 (1) −
4.81	28	0.10 (1) −	0	0	0.10 (1) −

^a^ Statistical diagnoses [33,34]: + (positive) and − (negative). Significance levels α = β = 0.05, one-sided test without Bonferroni correction; ^b^ 1 mM sinigrin = 397.46 µg·mL^−1^ sinigrin.

**Figure 2:**
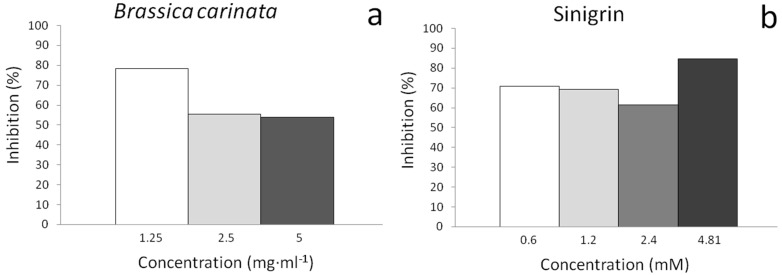
Inhibition effects of: (**a**) *B. carinat*a and (**b**) sinigrin, against H_2_O_2_ (0.12 mM) as genetic damage inductor. Correspondence: 1 mM sinigrin = 397.46 µg·mL^−1^ sinigrin.

Both samples showed a high desmutagenic and recombinogenic potency always producing the total mutation rates below the negative control values (Table 3). An inverse dose effect was also observed for *B. carinata* and sinigrin samples so, the lowest concentrations assayed for plant samples were more antigenotoxic than those of higher (78.46% clone inhibition) but, for sinigrin samples this was reversed: the highest antigenotoxic effect corresponded to a sinigrin concentration of 4.81 mM with a percentage of inhibition of clone formation of 84.61% (Figure 2). This healthier beneficial effect of sinigrin at higher concentrations is in agreement with the survival and genotoxicity experiments in which its positive effect was superior to those of *B. carinata*. Unlike sinigrin, the effects of *B. carinata* samples are the result of a complex mixture with a large number of different compounds with different effects. Further studies will be required in order to elucidate the underlying *B. carinata* and sinigrin protective mechanism. Nevertheless, *B. carinata* antimutagenic properties have been demonstrated.

Other *Brassica* spp. like *B. oleracea* L. var. *acephala* (kale) present the same effect. The leaf extract of these plants has neither genotoxic nor clastogenic activity in cells of mice [39] but a strong antigenotoxic effect. This *Brassica* variety presents a similar GLS profile to *B. carinata*, both with sinigrin as major leaf GLS [40] and best candidate responsible of this DNA protective effect.

### 2.3. In Vitro Assays

The promyelocytic cell line HL60 has been selected as a model on a big variety of substances candidates to be used as anticarcinogens and has proved to be a robust test system for pilot screening experiments [41]. In the case of GLS breakdown products, different HL60 cell lines have been used to prove the anticarcinogenic properties of diverse ITC groups resulting AITC the most effective arresting cell cycle [42]. Now, we have used this system to determine the antitumor properties of the plant *B. carinata* and specifically, its major GLS sinigrin by measuring the relative inhibitory capacity of tumor growing in HL60 cells. Our experiments have evidenced that *B. carinata* plant possesses antiproliferative properties and highlight the use of this plant in cancer chemoprevention.

**Figure 3:**
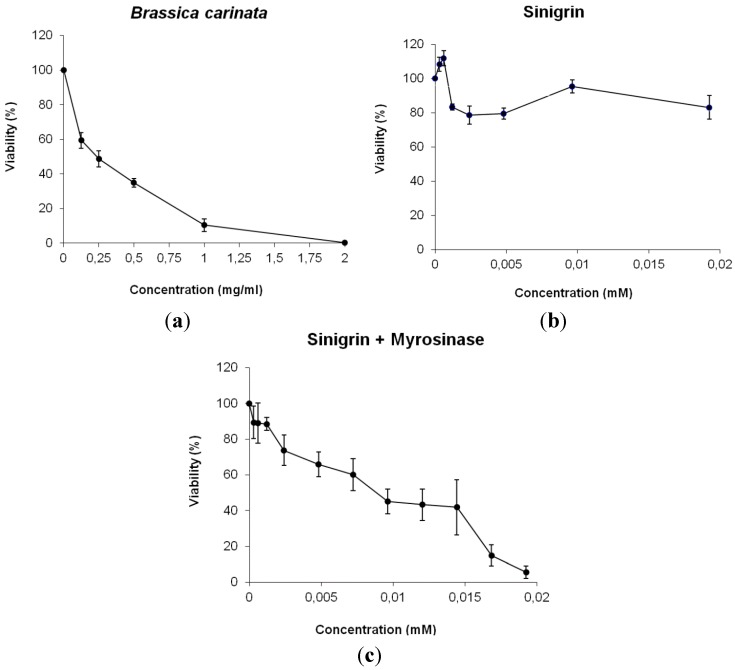
Survival of HL60 cultures treated with different concentrations of: (**a**) *B. carinata*; (**b**) sinigrin; (**c**) sinigrin + myrosinase. Curves are plotted as percentages with respect to the control counted from at least three independent experiments (mean ± SD). Correspondence: 1 mM sinigrin = 397.46 µg·mL^−1^ sinigrin.

The results on Figure 3 represent the relative growing rate of HL60 cultures (expressed as cell survival) with different concentrations of *B. carinata* and sinigrin respect to their concurrent control cultures. *B. carinata* results showed a dose-response curve with a high tumoricide activity in HL60 cells (IC_50_ value of 0.28 mg·mL^−1^). However, the antigenotoxic potency of sinigrin did not correlate with its null antiproliferative activity. The reason of this lack of cytotoxicity resides in the metabolic process by which GLSs produce their anticarcinogenic effect. In this sense, the fact that GLS sinigrin only acts as a cytotoxic agent when is hydrolysed by myrosinase enzyme revealing this mechanism.

Under natural conditions, GLSs and myrosinases are stored in separate compartments in plant tissues and different process like feeding by insect, animals or, in human case, food processing can put them into contact. Then, plant tissue disruption induce bioactivation of GLSs, putting myrosinases and their GLS substrates into contact, and GLS hydrolysis results in the formation of their biologically active products. To achieve this, our experiments performed using *D. melanogaster* individuals did not require more sample process that adds directly the plant or the bioactive compound to food waiting for their oral ingestion because of the presence of myrosinase in plant treatments or because myrosinase activity occurs normally during digestion and in the colon by microbial activity (sinigrin treatments) [6]. Contrarily, cytotoxicity assays with HL60 cell lines required the addition of an external myrosinase to sinigrin solution as a necessary step in order to produce its hydrolysis and antiproliferative activity (Figure 3C) because the addition of single sinigrin to cell medium did not produce expected effect by reducing cell proliferation (Figure 3B). So that, sinigrin produced antiproliferative activity in HL60 culture only after its hydrolysis by the enzyme myrosinase (hydrolysed sinigrin IC_50_ = 2.71 µM). In this sense, the presence of constitutive myrosinase in *B. carinata* plant samples justifies the antiproliferative activity found in the present research (Figure 3A). This activity also shows that lyophilized plants do not affect myrosinase function and conserved it intact, unlike other processing methods [6] although the combined treatment of plant and myrosinase has not been assayed. As a dependent temperature enzyme, myrosinase activity is favoured by low heat process up to 30 °C (*in vitro* HL60 experiment temperature) [43], facilitating the total GLS hydrolysis and the apparition of their healthy properties. Authors suggest that the human body temperature should be high enough for a proper GLSs digestion and nutrient intake. In addition, another plant proteins and active compounds will be protected to maintain intact plant health effects.

Others authors have found the same chemopreventive effect of *Brassica* spp. in certain tumor types by ceasing culture growth due to GLSs hydrolyzed into ITCs [16,44,45]. All these research has focused on the anti-carcinogenic activity of GLSs and ITCs as responsible compounds and that their intake and posterior digestion allow this process. In this sense, other authors [46] founded that sinigrin is able to inhibit induced hepatocarcinogenesis in rats when it is orally given in a diet containing 1.2 mg sinigrin·mg^−1^ food. This antiproliferative activity has also been shown in human *in vitro* models as HT29 colorectal tumor cells [47] and SK-Hep1 human hepatoma cells [48]. Our results are consistent with these researches because sinigrin is not cytotoxic but its hydrolysis products are. Further studies using each individual breakdown product of sinigrin can be conducted to elucidate the process whereby *B. carinata* induces the antitumor activity.

In conclusion, our results not only support the health benefits for humans of *B. carinata* plant in daily intake as a recommended vegetable to include in human diet, but also present sinigrin as precursor of AITCs the bioactive chemicals responsible of this effect and break-down products of sinigrin hydrolysis by myrosinase enzyme.

## 3. Experimental Section

### 3.1. Plant Material and Chemicals

The analysed genotype of Ethiopian mustard (*Brassica carinata* A. Braun) Bc-IASC1 is part of a *Brassica* germplasm collection of the Institute of Sustainable Agriculture (IAS-CSIC, Córdoba, Spain). This line was selected because of its drought resistance and good agronomic performance in previous studies carried out under rainfed conditions typical of the Mediterranean ecosystem [11]. Plants were cultivated in an experimental orchard at the IAS (37°52′N 4°46′W) wherein the climate is typically Mediterranean, with an average annual rainfall of 650 mm. The soil is deep and sandy-loam, classified as a Typic Xerofluvent. Leaves of five plants were harvested when they reached the stem elongation stage corresponding to code 49 of the BBCH scale [49], weighed, frozen (24 h at −80 °C) and lyophilized with a freeze-drier Telstar model Cryodos-50 (Telstar, Terrasa, Spain). After lyophilisation, dry material was weighed again, grounded in a Janke and Kunkel Model A10 mill (IKA-Labortechnik, Staufen, Germany) for about 20 s, mixed and conserved at room temperature and darkness to preserve their properties until use.

Sinigrin (C_10_H_16_KNO_9_S_2_·H_2_O) and myrosinase (EC 3.2.1.147) were purchased from Sigma-Aldrich (St. Louis, MO, USA) and glucotropaeolin (C_14_H_18_KNO_9_S_2_) from Phytoplan (www.phytoplan.de; Heidelberg, Germany).

### 3.2. HPLC

The GLS composition of *B. carinata* samples was determined by High Performance Liquid Chromatography (HPLC) [50]. About 100 mg of DW of sample was weighed and a two-step glucosinolate extraction was carried out in a water bath at 75 °C to inactivate myrosinase. In the first step the sample was heated for 15 min in 2.5 mL 70% aqueous methanol and 200 µL 10 mM glucotropaeolin (benzyl-GLS) as internal standard. A second extraction was applied after centrifugation (5 min, 5000 g) by using 2 mL of 70% aqueous methanol. One mL of the combined glucosinolate extracts was pipette onto the top of an ion-exchange column containing 1 mL Sephadex DEAE-A25 in the formiate form. Desulphation was carried out by the addition of 75 µL of purified sulphatase (E.C. 3.1.6.1, type H-1 from Helix pomatia) (Sigma-Aldrich, St. Louis, MO, USA) solution. Sulphatase was purified according to the ISO protocol (ISO 9167-1, 1992) [51]. Desulphated glucosinolates were eluted with 2.5 mL (0.5 mL × 5) Milli-Q (Millipore, Merck, Damstadt, Germany) ultra-pure water and analysed with a Model 600 HPLC instrument (Waters, Milford, MA, USA) equipped with a Model 486 UV tunable absorbance detector (Waters) at a wavelength of 229 nm. Separation was carried out by using a Lichrospher 100 RP-18 in Lichrocart 125-4 column, 5 µm particle size (Merck, Damstadt, Germany). HPLC solvents and gradient were according to the ISO protocol (ISO 9167-1, 1992). The HPLC chromatogram was compared to the desulpho-glucosinolate profile of three certified reference materials recommended by U.E. and ISO (CRMs 190, 366 and 367) analysed under the same conditions except for the use of sinigrin (2-propenyl-GLS) as internal standard [51]. The amount of each individual glucosinolate present in the sample was calculated by mean of the internal standard, and expressed as µmol·g^−1^ of DW. Data were corrected for UV response factors for different types of glucosinolates (ISO 9167-1, 1992). Data results were analyzed using the work station Waters Millenium 32 Chromatography Manager Software.

### 3.3. Fly Stocks and Crosses

The genotoxic and antigenotoxic activity of lyophilized leaves of *B. carinata* and sinigrin were evaluated by the *Drosophila* Somatic Mutation and Recombination Test (SMART) [52]. *Drosophila* is a holometabolous animal which *in vivo* experiments permit to make analogies with humans due to the high percentage of homologue genes in common with human [53]. We have selected the *D. melanogaster* SMART as a well-known eukaryotic assay that represents a rapid and economic way to evaluate the genotoxicity and antigenotoxicity of single compounds and complex mixtures [54]. Both characteristics make this test an optimal protocol in order to test molecules and complex mixtures with health benefices for humans. Two different *D. melanogaster* strains carrying visible wing genetic markers on the left arm of chromosome 3 were used: the *flare* (*flr*) strain *flr*^3^/*ln* (*3LR*) *TM3*, *Bd^s^* and the multiple wing-hair (*mwh*) strain *mwh*/*mwh* [55]. These markers were selected due to their position in the *Drosophila* genome, covering a wide portion of one of their four chromosomes. Specifically, they affect the wing imaginal disks which present more cells with a uniform growth during larvae development than other *Drosophila* imaginal disks. This fact makes possible to detect easily a high number of mutations, somatic recombinations and disjunctions produced in the *Drosophila* DNA during all treatment periods by wing microscopic observation. In this sense, these markers are present in other genetic background (strains) but crosses produce mutations with a basal mutation rate too high and disturbing hair anomalous pattems. The marker flare (*flr*^3^, *3_38.3*) is a recessive mutation which produces individual wing hairs that are malformed. The *flr*^3^ allele is a zygotic recessive lethal, which is maintained in the strain over the balancer chromosome *TM3* (*TM3*, *Bd^S^*: Third Multiple 3, Beaded-Serrate). The marker multiple wing hairs (*mwh*, *3_0.3*) is a recessive mutation that is viable in homozygous flies, producing multiple hairs per cell instead of the wild type single-hair trichome. Two types of crosses were used: the standard cross with *flr*^3^/*TM3*, *Bd^S^* females mated to *mwh*/*mwh* males and the reciprocal cross.

### 3.4. Toxicity, Genotoxicity and Antigenotoxicity Studies

Hybrid eggs from crossing optimally fertile flies were collected over an 8-h period. Emerged larvae, 72 ± 4 h later, were cleaned up from remaining feeding medium, using sterile distilled water, and subsequently were transferred into treatment vials. These vials contained tested conditions which consisted of 0.85 g of *Drosophila* Instant Medium (Formula 4–24, Carolina Biological Supply, Burlington, NC, USA) wetted with 4 mL of a mixture of distilled-sterile water and the appropriate concentration of lyophilized plant or the single compound sinigrin.

Genotoxicity/antigenotoxicity tests were performed following the standard protocol [52]. Antigenotoxicity tests were carried out by mixing the genotoxin hydrogen peroxide (H_2_O_2_, Sigma-Aldrich) with the plant or single compound in appropriate concentrations.

The groups consisting of approximately 100 larvae each were: (i) negative control (distilled water); (ii) mutagenic positive control (H_2_O_2_ 0.12 M); (iii) 3 vials with increasing concentrations of *B. carinata* (1.25, 2.5, and 5 mg·mL^−1^); and (iv) 4 vials with increasing concentrations of sinigrin (0.6, 1.2, 2.4 and 4.8 mM). Sinigrin concentrations were chosen on the basis of the sinigrin content of the *B. carinata* sample used in this study.

Larvae were fed on both treated and control mediums until pupation (about 48 h). After emergence, resulting adult flies were collected from the treatment vials, sacrificed under CO_2_ narcotization and stored in a 70% ethanol solution in sterile water.

Transheterozygous wings (*mwhflr^+^*/*mwh^+^ flr*^3^) were mounted and wing hair mutations (spots) were scored. Both dorsal and ventral surfaces of the wings containing 22,000 cells were screened under a photonic microscope (Nikon, Amsterdam, Netherlands) at 400× magnification for the occurrence of individual spots (*mwh* or *flr* phenotype) or twin spots (*mwh* clone adjacent to *flr* clone). Small individual spots with one or two cells exhibiting the *mwh* phenotype corresponded to gene mutation and somatic recombination between the two marker genes occurring during the last mitotic rounds in the imaginal discs of the larvae. Large individual spots with three or more cells showing *mwh* or *flr* phenotypes corresponded to mutational events occurring earlier during larvae development. Twin spots with two juxtaposed clones corresponded uniquely to recombination events between the *flr*^3^ gene and the centromere.

### 3.5. Cell Culture and Treatments

The human leukaemia cell line HL60 (promyelocitic cells) was supplied by Dr. José M. Villalba Montoro (Department of Cell Biology, University of Cordoba, Cordoba, Spain). HL60 cells were routinely grown in suspension in RPMI-1640 medium (ThermoFisher, Invitrogen, Madrid, Spain). This medium was supplemented with an antibiotic antimycotic solution 100× (A5955, Sigma-Aldrich, St. Louis, MO, USA) L-glutamine 200 mM (Sigma-Aldrich) and 10% heat-inactivated foetal bovine serum (Linus) in a 5% CO_2_ humidified atmosphere at 37 °C using a CO_2_ Incubator (Shellab, Cornelious, OR, USA). HL60 cells were subcultured every 2–3 days to maintain logarithmic growth and they were allowed to grow for 48 h before use. Cultures were plated at a density of 12.5 × 104 cells·mL^−1^ in 40 mL culture bottles (25 cm^2^).

The cytotoxic activity of the treatments was measured as growing inhibition or decreased viability on the human promyelocytic leukaemia cell line HL60. For measuring the cytotoxic effect of tested samples a general protocol [56] was modified by us. Cells were placed in 12 well culture plates (1 × 10^5^ cells·mL^−1^; final volume per well was 2 mL) and treated with different filtered (Millipore “non-pyrogenic”, “sterile-R”, 0.2 μm filter) RPMI solutions with the selected concentrations of *B. carinata* (0.125, 0.25, 0.5, 1 and 2 mg·mL^−1^), sinigrin (0.3, 0.6, 1.2, 2.4, 4.8, 9.6 and, 19.25 mM) and the mixture of sinigrin (0.3, 0.6, 1.2, 2.4, 4.8, 7.2, 9.6, 12, 14.4, 16.8 and 19.25 mM) + myrosinase. Cells were counted each 24 h for 7 days. Tested concentrations were calculated based on those used for genotoxicity assays to equal the range of tested doses. The myrosinase-catalysed hydrolysis products of sinigrin solution were prepared dissolving the intact sinigrin in RPMI medium (3.2 mg·mL^−1^) in a volume of 5 mL (stock solution). After total dissolution, myrosinase enzyme was added at a concentration of 5 mM. This stock solution (sinigrin + myrosinase) was homogenized by shaking and incubated at 30 °C for 30 min to activate the enzyme and produce sinigrin hydrolysis [56]. Then, sinigrin hydrolysed solution was diluted to the desired concentrations and applied to cell cultures. An enzyme control solution (without sinigrin) containing only RPMI medium with myrosinase was prepared and used in the same conditions in order to verify that enzyme do not affect cells when compared with negative control (untreated culture).

#### Cell Viability Assay

Cell viability was determined by the Trypan Blue dye exclusion test (Sigma-Aldrich). Cells were counted by adding an aliquot of 10 μL of the culture to 10 μL of the Trypan Blue dye solution. The mix was counted under a light inverted microscope (AE30/31, Motic Spain SLU, Barcelona, Spain) using a Neubauer chamber. Non-viable cells stained purple-violet, whereas viable cells remained unstained.

### 3.6. Statistical Analysis

Toxicity (T) was determined as percentage of survival adults with respect to 450 untreated 72 h old larvae from three independent experiments [31]:

T = (N° of emerging individuals in treatment/N° of emerging individuals in the negative control) × 100(1)

For the evaluation of genotoxic effects, the frequencies of spots per fly of each treated series were compared to the concurrent negative control for each class of mutational clone. A multiple-decision procedure was used to categorize results as positive, positive, inconclusive, or negative [33]. Statistical analyses were carried out for single (a small single spot corresponding to one or two cells exhibiting the *mwh* phenotype), large (a large single spot corresponding to three or more cells showing *mwh* or *flr*^3^ phenotypes), twin (a large single spot corresponding to three or more cells showing *mwh* or *flr*^3^ phenotypes) and total number of spots recovered. Inconclusive and positive data were evaluated by the non-parametric U test of Mann, Whitney and Wilcoxon [34]. The inhibition percentage (IP) was calculated as follows [57]:

IP = (genotoxin alone − sample + genotoxin) × 100/(genotoxin alone)(2)

For the evaluation of cytotoxic effects, after each culture incubation period, a growth curve was established and IC_50_ values (concentration of tested compound causing 50% inhibition of cell growth) were estimated. Viability curves of leukaemia cells were expressed as percentage of survival with respect to controls at 72 h of growth and plotted as mean viability ± standard error of at least three independent replicas for each treatment and concentration. Statistical analyses were performed using a Microsoft 2007 Excel spreadsheet. The non-parametric U test of Mann, Whitney and Wilcoxon were assessed with the SPSS Statistic 17.0 software (SPSS, Inc., Chicago, IL, USA).
